# Equitable Access to Mental Health Services in Brazil

**DOI:** 10.1192/j.eurpsy.2025.1653

**Published:** 2025-08-26

**Authors:** M. A. Rodrigues

**Affiliations:** 1 Universidade Anhembi Morumbi, São Paulo, Brazil

## Abstract

**Introduction:**

Equitable access to mental health services is a challenge in Brazil, marked by socioeconomic and regional inequalities. Despite advances in policies such as psychiatric reform and the Psychosocial Care Network (RAPS), barriers persist, especially for vulnerable populations. Issues such as the unequal distribution of resources, stigma, and a lack of professionals impact both access and the quality of care.

**Objectives:**

To analyze the inequalities in access to mental health services in Brazil and identify strategies to promote equity.

**Methods:**

A systematic review was conducted using the PubMed, Scielo, and Lilacs databases, covering studies published between 2015 and 2023. The keywords used were “mental health access,” “health equity,” “Brazil,” and “mental health inequalities.” Inclusion criteria focused on studies addressing access to mental health services in Brazil, highlighting barriers, facilitators, and strategies to promote equity. Data were qualitatively synthesized to identify trends and challenges.

**Results:**

The review revealed significant disparities in access to mental health services, influenced by socioeconomic, geographic, and cultural factors. Remote regions, such as the North and Northeast, suffer from a lack of infrastructure and specialized professionals. While urban centers offer more services, rural areas face considerable limitations. The unequal distribution of professionals, such as psychiatrists and psychologists, exacerbates these inequalities. Stigma surrounding mental illness and a lack of awareness further hinder help-seeking behavior. Vulnerable populations, including Indigenous peoples and residents of favelas, face additional barriers such as discrimination and a lack of culturally adapted services.

The Unified Health System (SUS) provides a model of universal access but faces challenges related to funding, infrastructure, and capacity to meet the growing demand. Despite the efforts of RAPS, many services are overwhelmed, resulting in long waiting times and insufficient care. Strategies like integrating mental health into primary care have shown effectiveness but require more support and expansion.

**Conclusions:**

Equitable access to mental health care in Brazil is impacted by various barriers, including regional disparities and stigma. To promote equity, it is crucial to invest in expanding the mental health care network, focusing on underserved areas and vulnerable populations. Effective policies should include professional training, stigma reduction, and culturally sensitive services. Integrating mental health services with primary care, strengthened by the SUS, is essential to ensure more accessible and comprehensive care.

**Disclosure of Interest:**

None Declared

